# Single‐nucleus multi‐ome profiling of epigenomic modifications and transcriptome characterization of heterogeneity in early and late‐stage Alzheimer’s disease

**DOI:** 10.1002/alz.085676

**Published:** 2025-01-03

**Authors:** Zechuan Shi, Sudeshna Das, Samuel Morabito, Nora Emerson, Negin Rahimzadeh, Nellie Kwang, Elizabeth Head, Vivek Swarup

**Affiliations:** ^1^ Institute for Memory Impairments and Neurological Disorders, Irvine, CA USA; ^2^ University of California, Irvine, Irvine, CA USA; ^3^ Institute for Memory Impairments and Neurological Disorders (MIND), Irvine, CA USA; ^4^ Mathematical, Computational and Systems Biology (MCSB) Program, UC Irvine, Irvine, CA USA; ^5^ The UC Irvine Institute for Memory Impairments and Neurological Disorders (UCI MIND), Irvine, CA USA

## Abstract

**Background:**

Alzheimer’s disease (AD), characterized by tau lesions and amyloid plaques, has traditionally been investigated within the cortical domain. Recent neuroimaging studies have implicated micro‐ and macrostructural abnormalities in cortical layers during the progression of AD. While examinations from diverse brain regions have contributed to comprehending the regional severity, these approaches have constrained the ability to delineate cortical alterations in AD. Our study employed a single‐cell resolution approach to characterize transcriptomic and epigenomic alternations in the human brain affected by the disease.

**Method:**

We conducted a comprehensive analysis utilizing snRNA‐Seq and single‐nucleus epigenomic profiling on samples obtained from more than 2 million cells over 100 individuals with early and late‐stage AD cases, along with controls. Four samples were procured from each participant, derived from the human pre‐frontal cortex (PFC) and temporal cortex (TCX). Our analysis utilized multi‐ome profiling, which simultaneously profiles gene expression and epigenomic changes. Data processing utilized Scanpy and Signac for gene expression and histone processing. Cicero identified cis‐regulatory elements (cCREs), overlaid with GWAS variants, creating a genome‐wide map. Transcription factor (TF) footprinting was done for cell types and disease‐associated subclusters.

**Result:**

UMAP and Leiden clustering on batch‐corrected data identified discrete cell‐type clusters from snRNA‐Seq and additional multi‐ome profiling. Enhancer‐promoter links were identified, constructing disease‐enriched, region‐specific, and shared TF regulatory networks in AD. Pseudotime trajectory analysis revealed changes from early to late‐stage AD.

**Conclusion:**

The single nucleus multi‐ome data produced in this study represents a valuable asset for the AD research community. Compared to bulk ATAC‐seq, our single‐nucleus epigenomic data provides precise insights into active and repressive activities. The study illuminates crucial TFs and their roles in AD, offering insights into cell‐specific and region‐specific mechanisms in AD pathology. Experimental validation underscores the functional relevance of the targeted TF in AD, highlighting its potential medical significance.

